# Cognitive Investments in Academic Success: The Role of Need for Cognition at University

**DOI:** 10.3389/fpsyg.2017.00790

**Published:** 2017-05-16

**Authors:** Julia Grass, Alexander Strobel, Anja Strobel

**Affiliations:** ^1^Personality Psychology and Assessment, Department of Psychology, Chemnitz University of TechnologyChemnitz, Germany; ^2^Differential and Personality Psychology, Department of Psychology, Technische Universität DresdenDresden, Germany

**Keywords:** need for cognition, academic success, satisfaction with one's studies, investment traits, academic performance

## Abstract

Previous research has shown that Need for Cognition (NFC), the individual tendency to engage in and enjoy cognitive endeavors, contributes to academic performance. Most studies on NFC and related constructs have thereby focused on grades to capture tertiary academic success. This study aimed at a more comprehensive approach on NFC's meaning to success in university. We examined not only performance but also rather affective indicators of success. The current sample consisted of 396 students of different subjects with a mean age of 24 years (139 male). All participants took part in an online survey that assessed NFC together with school performance and further personality variables via self-report. Success in university was comprehensively operationalized including performance, satisfaction with one's studies, and thoughts about quitting/changing one's major as indicators. The value of NFC in predicting tertiary academic success was examined with correlation analyses and path analysis. NFC significantly correlated with all success variables with the highest correlation for study satisfaction. Path analysis confirmed the importance of NFC for study satisfaction showing that NFC had a significant direct effect on study satisfaction and via this variable also a significant indirect effect on termination thoughts. This study clearly indicates that NFC broadly contributes to the mastery of academic requirements and that it is worthwhile to intensify research on NFC in the context of tertiary education.

## Introduction

In decades of research, comprehensive studies have shown that intelligence as maximum cognitive performance (see Ackerman and Heggestad, 1997) is one if not the most relevant predictor concerning academic achievement and success in educational and work contexts, respectively (Schmidt and Hunter, 1998; Deary et al., 2007; Strenze, 2007; Poropat, 2009). On the other hand, the *typically* invested amount of cognitive effort, as it is described by intellectual investment traits, has not been considered to the same degree (von Stumm et al., 2011b). Investment traits are characterized as “stable individual differences in the tendency to seek out, engage in, enjoy, and continuously pursue opportunities for effortful cognitive activity” (von Stumm et al., 2011a, p. 225) that is, they determine how individuals invest their cognitive resources, how they deal with cognitively challenging material and how they enjoy being involved in such tasks (Ackerman and Heggestad, 1997). Recently, a growing field of research examined the relevance of investment traits pointing to their influence concerning intellectual development, intelligence, and academic achievement (e.g., Preckel et al., 2006; von Stumm et al., 2011b; von Stumm and Ackerman, 2013). Accordingly, in this article, we address the investment trait *Need for Cognition* (*NFC*), a “stable individual difference in people's tendency to engage in and enjoy effortful cognitive activity” (Cacioppo et al., 1996, p. 197) and its relation to academic success. While often only defined by achievement in terms of grades (e.g., grade point average; GPA) or similar aspects (Trapmann et al., 2007b), we additionally focus on another essential aspect of academic success that has been sparsely investigated: the satisfaction with one's studies (Trapmann et al., 2007a,b). Different articles have hinted at the necessity to go beyond the GPA in order to capture the complex meaning of success (e.g., Rindermann and Oubaid, 1999; Robbins et al., 2004; Trapmann et al., 2007b). Therefore, this study considers different success indicators to give a comprehensive view on the topic.

As stated above, NFC describes individual differences in the tendency to approach cognitively demanding situations and to enjoy elaborated thinking (Cacioppo and Petty, 1982). It has a large conceptual overlap with other investment traits like Typical Intellectual Engagement (TIE; Goff and Ackerman, 1992; see Mussel, 2010), is closely related to the Big-Five facet Openness to Ideas (Fleischhauer et al., 2010) and can (together with TIE) be regarded as a core aspect of intellectual investment (von Stumm and Ackerman, 2013). NFC was found to have small to medium positive relations to Conscientiousness, Emotional Stability, and goal-directed behavior (Fleischhauer et al., 2010). Its associations with intelligence do not exceed a medium size (e.g., Cacioppo et al., 1996; Fleischhauer et al., 2010; Hill et al., 2013). Concerning academic performance, previous studies found NFC to be weakly to moderately correlated with college students' performance (Cacioppo and Petty, 1982; Tolentino et al., 1990; Richardson et al., 2012). In school contexts, NFC has been shown to possess predictive validity over and above other non-cognitive constructs commonly investigated in educational research (Preckel et al., 2006; Meier et al., 2014) pointing to its importance in other academic contexts, too. The closely related TIE meta-analytically predicted academic performance directly and additionally to Conscientiousness and intelligence with a path parameter of 0.20 (ρ = 0.33, von Stumm et al., 2011b). The few reviews including NFC report comparable correlations for NFC and academic performance (ρ = 0.17–0.22, Richardson et al., 2012; von Stumm and Ackerman, 2013). Whereas, achievement motivation and other traditional motivational constructs have been examined more often (Robbins et al., 2004; Richardson et al., 2012), according to von Stumm and Ackerman (2013), at the time of their review, only 12 studies provided data to compute relations between NFC and academic performance.

However, to consider academic success only as getting good marks and successfully passing the examinations would be short-sighted. Different studies have hinted at the necessity to go beyond grades in order to capture the complex meaning of success (e.g., Rindermann and Oubaid, 1999; Robbins et al., 2004; Trapmann et al., 2007b). Thereby different indicators assess different aspects (Chamorro-Premuzic and Furnham, 2003; Robbins et al., 2004) but are interrelated: For instance, job satisfaction was shown to be medium positively related to better performance (Judge et al., 2001) and negatively to increased intentions to leave (Hellman, 1997). Hence, academic success also depends on the satisfaction of students concerning their studies or, broadly spoken, on their well-being in the context of their studies. NFC has been shown to be positively associated with affective variables like self-esteem (Cacioppo et al., 1996) and affective adjustment (Bertrams and Dickhäuser, 2012) that are likely to support adaptive reactions to academic demands and to challenging academic situations. Furthermore, different studies have hinted at associations between NFC and aspects of satisfaction: In a first study dealing with affective outcomes it was found that NFC was associated with higher life satisfaction during the college years (Coutinho and Woolery, 2004), which should be applicable to satisfaction within a particular domain, too (Lent et al., 2005). For individuals with higher NFC-scores, perceptions of higher complexity were found to lead to more elaborated processing (See et al., 2009) and to enhanced job satisfaction (Park et al., 2008). Surely, the requirements of university education as the highest educational track can be regarded as complex, that is, high-NFC-individuals should feel better in such a cognitively challenging environment.

However, up to now relations of NFC to well-being have been examined in a more general way (life satisfaction; Coutinho and Woolery, 2004), using variables underlying satisfaction or well-being (e.g., self-esteem; Cacioppo et al., 1996; Bertrams and Dickhäuser, 2012) or regarding different contexts (e.g., job-related context; Park et al., 2008). So on the one hand, there are only a few studies on affective implications of NFC in general and on the other hand, there is no research at all on direct relations of NFC to academic satisfaction. Taken together, a positive relation of NFC to students' satisfaction with their studies has not been directly examined yet and can only be assumed due to the few studies that have already reported associations of NFC with satisfaction in other contexts as well as to probably underlying variables of affective adjustment.

As mentioned above, intelligence is an important and established predictor of academic performance (e.g., Deary et al., 2007; Poropat, 2009). However, often it is costly to assess intelligence in all applicants, so indicators of previous academic performance are alternatively considered (Trapmann et al., 2007b). At German universities, selection processes often rely only on the GPA of the university entrance diploma (Rindermann and Oubaid, 1999). Supporting this practice, meta-analyses have shown prior academic performance as an important predictor for educational and occupational levels (Strenze, 2007) and for academic achievement (Trapmann et al., 2007b). Recent reviews reported average associations of school grades with academic achievement in university ranging from about *r* = 0.25 to 0.40 (Robbins et al., 2004; Trapmann et al., 2007b; Richardson et al., 2012).

Furthermore, there are many findings concerning broader personality variables and their relation to academic success (Robbins et al., 2004; Poropat, 2009; Richardson et al., 2012). Two reviews found Conscientiousness to be the only Big-Five factor (Goldberg, 1990) that is able to incrementally predict tertiary academic performance over and above cognitive abilities (Trapmann et al., 2007a; Poropat, 2009). Likewise, in a recent meta-analysis, intelligence, Conscientiousness, and TIE were direct, correlated predictors of academic performance (von Stumm et al., 2011b). In turn, Neuroticism was strongly negatively associated with satisfaction with one's studies (Trapmann et al., 2007a).

### The current study

As outlined above, previous research suggests NFC to be of importance in predicting academic performance besides broader personality traits and cognitive ability but more empirical data is needed to support this claim. Furthermore, the operationalization of academic success is usually restricted to performance disregarding other facets like satisfaction with one's studies. Just like that, research on NFC has focused rather on cognitive implications than on affective ones and respective evidence still needs to be enlarged. Thus, with the current study, we aimed to extend previous research on NFC and tertiary academic achievement by considering not only grades but different facets of success in university within one sample. As mentioned above, existing research on affective variables focused on other aspects of satisfaction (life satisfaction; Coutinho and Woolery, 2004) or on variables underlying important affective outcomes (e.g., self-control capacity; Bertrams and Dickhäuser, 2012). Therefore, the current study aimed at transferring former findings concerning affective implications of NFC to the university context using study-related indicators.

Accordingly, (1) We examined the zero-order correlations of NFC to different indicators of academic success. On the basis of Richardson et al. (2012) and von Stumm and Ackerman (2013), we expected small to moderate positive correlations for NFC with students' academic performance. Furthermore, we expected positive associations with satisfaction with one's studies: Enjoying effortful thinking should promote the enjoyment of the tasks that are necessary for completing one's university studies successfully. If higher NFC enhances satisfaction with one's studies, it should also decrease the frequency of thoughts about quitting or changing one's major (referred to as termination thoughts). We exploratory examined how NFC was related to self-reported reasons for such termination thoughts.

**Figure 1:**
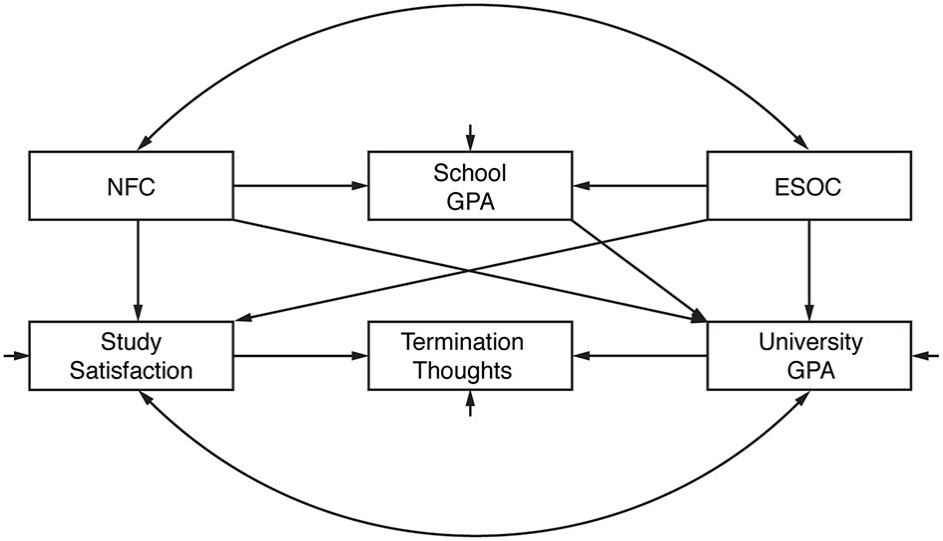
**Model underlying path analysis**. NFC, Need for Cognition. GPA, grade point average. ESOC, overall score of inverse Neuroticism (i.e., Emotional Stability), Openness, and Conscientiousness that should reflect personality characteristics beneficial for academic success.

(2) Furthermore, interrelations between variables, especially possible indirect effects, were examined using path analysis. Based on the body of literature outlined above, we expected that (a) NFC and relevant broader personality traits would be correlated, but would independently and positively impact on school GPA. In order to reduce model complexity with regard to relevant personality variables, we computed an overall ESOC score of inverse Neuroticism (i.e., Emotional Stability), Openness, and Conscientiousness that should reflect personality characteristics beneficial for academic success. We further assumed that (b) university GPA would also be influenced by NFC and ESOC, but also by school GPA, which is all the more likely as the latter is relevant for university admittance in a number of subjects. (c) Study satisfaction supposedly would be modulated by NFC and ESOC, but also by university GPA. Finally, we expected that (d) termination thoughts could arise from both lower university GPA and lower study satisfaction. The model is depicted in Figure 1.

## Materials and methods

### Procedure

This study was conducted online via EFS survey (Version EFS 10.5; QuestBack GmbH, 2015) with anonymous participation. At the beginning, all participants were informed about the topic of the study and gave written informed consent in accordance with the Declaration of Helsinki. Then they answered demographic questions and filled out all measures outlined below. Finally, participants were asked about disturbing influences and about the honesty of their responses. Participants were forced to answer most questions except for those related to their performance. The procedure was evaluated by the Ethics Committee of the Faculty of Behavioral and Social Sciences. It was not considered to require further ethical approvals and hence, as uncritical concerning ethical aspects according to the criteria^1^ used by the Ethics committee.

### Participants

Participants were recruited via email platforms of German universities, social media, and advertisements on the campus of a German university. A total of 407 participants responded to all instruments. Three of them stated they had answered dishonestly, and two partly showed no response variability; these five were excluded. We excluded additional five participants who were doing their PhD and one student who was in an orientation semester. Thus, the final sample included 396 participants (135 male, *M*_age_ 24.08 ± 4.72 years, range 18–49 years). They had currently been studying their major for 1–15 semesters (*M* = 4.42 ± 3.00). Most participants (37.9%) categorized their major subject of study as being in the field of humanities (e.g., educational sciences, sociology, English studies), 13–15% in mathematics/natural sciences, engineering sciences, and economics, respectively. 19.2% stated that they were studying psychology.

### Measures

#### Predictor variables

We assessed NFC with the German 16-item short scale (Bless et al., 1994). Responses were recorded on a 7-point rating scale from −3 (completely disagree) to +3 (completely agree) and summed.

Conscientiousness, Neuroticism, and Openness to Experience were assessed with the 21-item version of the Big Five Inventory (Rammstedt and John, 2005). Responses were rated on five levels of agreement from 1 (*strongly disagree*) to 5 *(strongly agree*). Responses were averaged per dimension.

We assessed previous academic performance by self-report of the GPA from the Abitur, that is, the German university entrance diploma. This measure is referred to as school GPA. Grades could range between 1 and 5. Grades were recoded so that 1 reflects failure and 5 indicates best performance.

#### Success in university

Participants reported their current GPA (referred to as university GPA) as well as their three best grades in their previous examinations. Grades could range between 1 and 5. Grades were recoded so that 1 reflects failure and 5 indicates the best performance^2^.

Satisfaction with one's studies was assessed with 12 items ^3^ that were based on Krapp et al. (1993) and Westermann et al. (1996). The 12-item measure is displayed in Table A1 in Supplementary Material. We combined aspects out of both instruments as we intended to assess aspects of satisfaction with one's studies that were to be found in different measures: satisfaction with the contents of one's studies (“I have chosen my current studies above all because of their interesting contents”), academic-related stress (“I often feel tired and tense because of my studies,” recoded) as well as general academic satisfaction/enjoyment (“I like to study”). Answers were scored on a 4-point rating scale from 1 (*totally untrue*) to 4 (*totally true*) and averaged.

Participants were asked whether they had ever thought of quitting their studies or changing their major domain of study (1 = *never* to 6 = *often*). When such termination thoughts occurred, the reasons were assessed with 12 categories including “lacking motivation,” “unappealing study contents,” and “pressure to perform.”

#### Control variables

We assessed age, gender, major subject of study, general field of study (e.g., humanities), and duration spent in the current major. Note, that additional variables were assessed for exploratory reasons that were outside the scope of this study (e.g., lay theories of intelligence and effort). Including these variables did not alter the results concerning NFC and study-related variables.

### Statistical analyses

Except for categorical variables, correlations were calculated with Spearman's rank coefficient because only a few variables were normally distributed (Kolmogorov–Smirnov tests, *p* > 0.05). As we conducted multiple comparisons, we applied the Bonferroni correction for 34 single comparisons, resulting in α = 0.05/34 ≈ 0.0015.

Path analysis was performed using RStudio (RStudio Team, 2016) with R 3.3.1 (R Core Team, 2016) and the package *lavaan* (Rosseel, 2012) as well as the additional packages *MissMech* (Jamshidian et al., 2014) and *psych* (Version 1.6.9; Revelle, 2016). In order to reduce violations of the assumption of multivariate normality, all variables were normalized in advance using Blom's formula (Blom, 1958). This resulted in an overall improvement in multivariate distribution characteristics as evaluated using QQ-plots and Mardia tests (original variables: *p*_skew_ < 1.7 ^*^ 10^−8^, *p*_kurtosis_ = 0.97; normalized variables: *p*_skew_ < 0.015, *p*_kurtosis_ = 0.13), but still, the data deviated from multivariate normality. However, maximum likelihood (ML) estimation penalizes this situation with worse fit indices (e.g., Curran et al., 1996; Yang and Liang, 2013), that is, if fit indices point to quite reasonable fit, one could expect that with multivariately normal data, even better model fit could have been achieved. Thus, the path model was fitted using ML estimation to allow for imputation of missing values using full-information maximum likelihood (FIML). Yet, to scrutinize the results, the analysis was repeated with diagonally weighted least squares (DWLS) estimation that can be considered the more appropriate method when the assumption of multivariate normality is violated (Li, 2016), but does not allow to impute missing values, resulting in a lower sample size. Model fit was evaluated using the Comparative Fit Index (CFI), the Root Mean Square Error of Approximation (RMSEA), and the Standardized Root Mean Square Residual (SRMR). Model fit was considered good for CFI > 0.95, RMSEA < 0.06, and SRMR < 0.08 (Hu and Bentler, 1999). Indirect effects were computed as products of the direct path coefficients for all pathways via which NFC might impact on the respective outcome variables: (a) NFC—school GPA—university GPA; (b) NFC—study satisfaction—termination thoughts; (c) NFC—university GPA—termination thoughts; (d) NFC—university GPA—study satisfaction; (e) NFC—study satisfaction—university GPA; (f) NFC—school GPA—university GPA—termination thoughts; (g) NFC—school GPA—university GPA—study satisfaction; and (h) NFC—school GPA—university GPA—study satisfaction—termination thoughts. For all direct and indirect effects, standard errors were determined using standard bootstrapping with 1,000 replicates.

## Results

Descriptive statistics and reliabilities of all instruments are displayed in Table 1.

**Table 1:** **Descriptive statistics and reliabilities of personality traits and success measures**.

**Score**	***M***	***SD***	**Min**	**Max**	***n***	**α^a^**
Need for cognition	17.06	12.69	−35.00	46.00	396	0.86
Personality factors						
Conscientiousness	3.55	0.72	1.50	5.00	396	0.73
Neuroticism	3.17	0.91	1.00	5.00	396	0.78
Openness to experience	3.97	0.70	1.80	5.00	396	0.71
School GPA^b^	3.83	0.64	2.30	5.00	395	–
Success in university						
University GPA^b^	3.85	0.67	1.00	5.00	367	–
Satisfaction with one's studies	3.01	0.42	1.50	3.92	396	0.80
Termination thoughts^c^	2.14	1.36	1.00	6.00	396	–

*N = 396. Different n due to selectively missing answers. Min, minimum value. Max, maximum value. GPA, grade point average*.

a *Internal consistency, Cronbach's alpha*.

b *Recoded: 1 = low performance, 5 = high performance*.

c *1 = never, 6 = often*.

### Relations between NFC and academic success

The correlations of all variables with NFC and outcome-measures of success in university are depicted in Table 2. NFC was significantly associated with all success measures comprising both, aspects of performance as well as satisfaction. As expected, we found a small positive correlation between NFC and university GPA (*r*_s_ = 0.19, *p* < 0.001). The strongest correlation concerning NFC could be observed for satisfaction with one's studies (*r*_s_ = 0.40, *p* < 0.001). Satisfaction with one's studies was moderately associated with better university GPA (*r*_s_ = 0.37, *p* < 0.001) and with less frequent termination thoughts (*r*_s_ = −0.48, *p* < 0.001). University GPA and termination thoughts were negatively related (*r*_s_ = −0.21, *p* < 0.001). Analyzing all correlations between NFC and the self-reported reasons of termination thoughts (*n* = 217), we found significant associations of *r*_pb_ = −0.23 (*p* = 0.001) with “not feeling that one belonged,” *r*_pb_ = −0.18 (*p* = 0.008) with a “lack of motivation,” and *r*_pb_ = −0.15 (*p* = 0.031) with a “perceived missing link between theory and practice.” NFC was not significantly associated (*p* > 0.05) with the remaining categories “pressure to perform,” “lacking success in one's studies,” “unappealing study contents,” “inadequate study conditions,” “interest in different (study) subjects,” “financing problems,” “bad occupational outlook/ future perspective,” “other personal issues,” and “dissatisfaction with study demands,” and a category of not classifiable answers. Each category was chosen by 5–85 participants; <50 statements were registered for the seven last-named categories (5–24 choices per category).

**Table 2:** **Correlations between the predictor variables, NFC, and success in university**.

	**Satisfaction with one's studies**	**Termination thoughts**	**University GPA**	**NFC**
Success in university				
Satisfaction with one's studies	–			
Termination thoughts	−0.48^**^	–		
University GPA	0.37^**^	−0.21^**^	–	
Predictors				
NFC	0.40^**^	−0.18^**^	0.19^**^	–
Gender^^b^^	0.10	−0.09	0.14^**^	−0.01^**^
Age	−0.10	−0.06	−0.11^*^	0.15^**^
Time in current major	−0.18^**^	0.08	−0.07	0.07^**^
School GPA	0.18^**^	−0.09	0.43^**^	0.16^**^
Conscientiousness	0.31^**^	−0.25^**^	0.28^**^	0.27^**^
Neuroticism	−0.21^**^	0.20^**^	−0.00	−0.25^**^
Openness to Experience	0.16^**^	0.01	−0.03	0.21^**^

*n = 367–396. Spearman's rank correlations except for the correlations with gender (point-biserial correlations). NFC, Need for Cognition; GPA, grade point average*.

*^a^Refers to the three best grades during the current studies*.

b *1, male; 2, female*.

* *p < 0.05*.

** *p < 0.01. **Bold:** Bonferroni-corrected significance level p < 0.0015*.

### Predicting academic success with NFC

**Figure 2:**
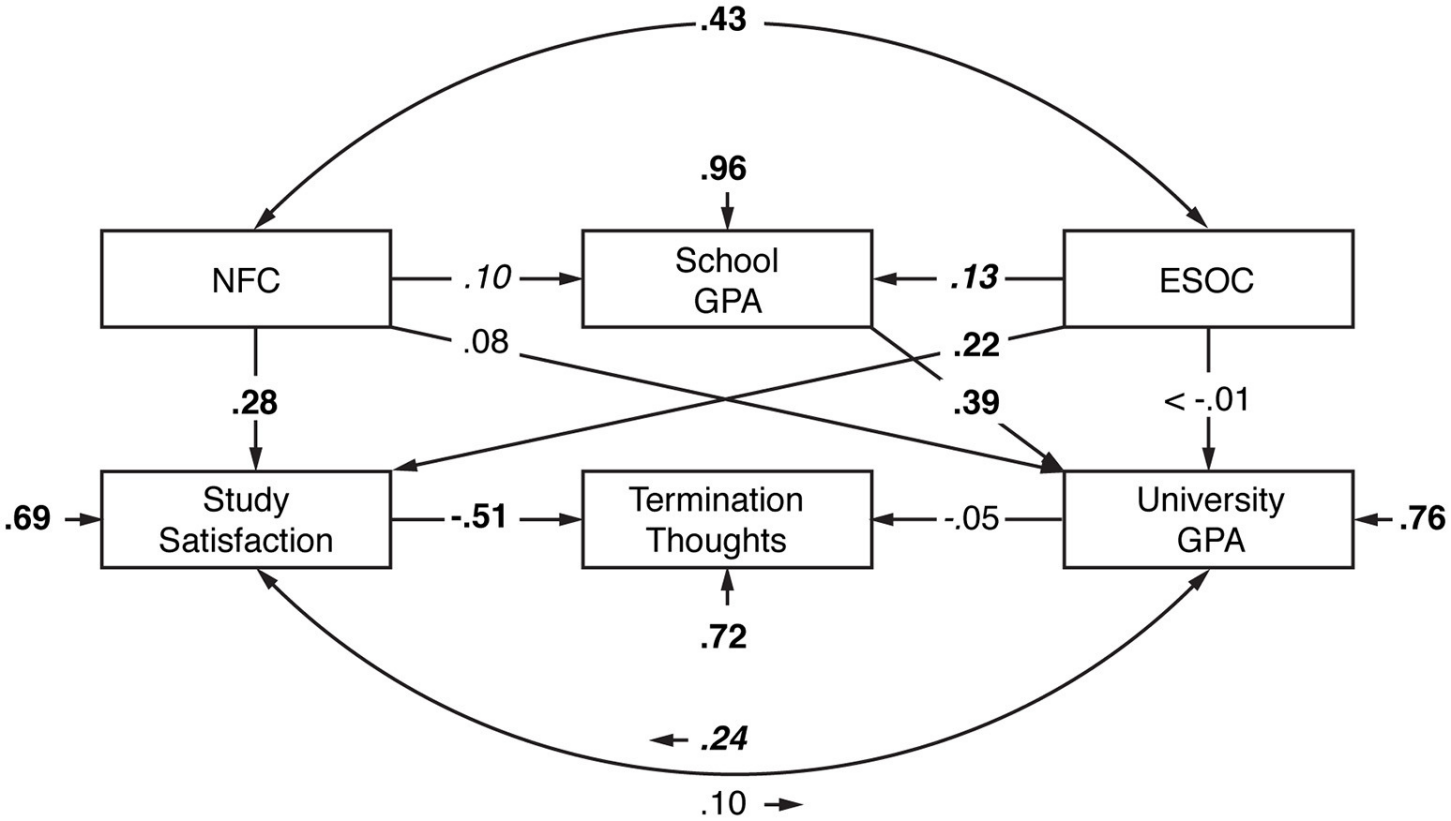
**Path model relating NFC to diverse academic outcomes**. NFC, Need for Cognition. GPA, grade point average. ESOC, overall score of inverse Neuroticism (i.e., Emotional Stability), Openness, and Conscientiousness that should reflect personality characteristics beneficial for academic success. Bold = *p* < 0.001, bold italic = *p* < 0.05, italic = *p* < 0.10. The indirect effect of NFC on Termination Thoughts via Study Satisfaction is significant (β = −0.14, *p* < 0.001). The criteria's variance explained by their predictors is 1 minus the respective error variances.

The fit of the path model in the total sample of *N* = 396 with FIML-imputed missing values was excellent (χ^2^ = 1.94, *df* = 3, *p* = 0.585, *CFI* = 1, *RMSEA* = 0.00 with 90% confidence interval 0.00–0.07, *SRMR* = 0.01). Figure 2 provides the standardized path coefficients for the direct paths. As expected, NFC and broad personality traits (ESOC) were correlated (*r* = 0.43, *p* < 0.001). The paths from NFC to study satisfaction (β = 0.28, *p* < 0.001) and from study satisfaction to termination thoughts were significant (β = −0.51, *p* < 0.001), as was the indirect path from NFC via study satisfaction to termination thoughts (β = −0.14, *p* < 0.001), while the other indirect paths involving NFC were insignificant (*p* ≥ 0.088). Further significant paths emerged from ESOC to school GPA (β = 0.13, *p* = 0.018) and to study satisfaction (β = 0.22, *p* < 0.001), from school GPA to university GPA (β = 0.36, *p* < 0.001), and from university GPA to study satisfaction (β = 0.24, *p* < 0.018; all other *p* ≥ 0.074). Using DWLS estimation of the model in those participants without missing values (*n* = 367), model fit was still excellent (χ^2^ = 0.77, *df* = 3, *p* = 0.856, *CFI* = 1, *RMSEA* = 0.00 with 90% confidence interval 0.00–0.05, *SRMR* = 0.01) and no essential changes in path coefficients and their significance occurred, indicating no influence of the estimation method on the stability of the results.

A model with all paths involving NFC fixed to zero–again estimated using ML/FIML–showed bad fit (χ^2^ = 41.52, *df* = 6, *p* < 0.001, *CFI* = 0.92, *RMSEA* = 0.12 with 90% confidence interval 0.09–0.16, *SRMR* = 0.06), and a χ^2^ differences test between this model and the model with free estimation of the path coefficients involving NFC was significant (χ^2^diff = 39.58, *df*_diff_ = 3, *p* < 0.001). This suggests that a model including NFC is superior to a model without NFC-related influences.

## Discussion

This study focused on the relevance of NFC for success in university, namely for academic performance, satisfaction with one's studies, and termination thoughts. Whereas, previous studies in this area focused mostly on performance measures, this study aimed at a broader perspective on NFC and academic success by additionally including subjective and more affective indicators of success in university. We examined direct relations as well as the predictive value of NFC likewise considering former school performance, Conscientiousness, Neuroticism, and Openness to Experience. NFC was positively associated with academic performance, with satisfaction, and with less termination thoughts. Remarkably, satisfaction with one's studies showed the strongest, medium-sized association with NFC. Path analysis confirmed the importance of NFC for study satisfaction showing that NFC had a significant direct effect on study satisfaction and via this variable also a significant indirect effect on termination thoughts.

### Direct relations

#### Performance

As expected, we found a small correlation of NFC with university GPA (*r*_s_ = 0.19), which is quite comparable to the average correlation found by Richardson et al. (2012) and von Stumm and Ackerman (2013). In our data, associations of university GPA with school GPA (*r*_s_ = 0.43) and Conscientiousness (*r*_s_ = 0.28) were (slightly) higher but still comparable to meta-analytic coefficients reported by Richardson et al. (2012).

#### Satisfaction

The focus of our study was to extent previous research by including rather affective measures of success as, for instance, satisfaction. We found the strongest relation of NFC with satisfaction with one's studies (*r*_s_ = 0.40). The correlation between satisfaction with one's studies and Conscientiousness was weaker (*r*_s_ = 0.31), followed by Neuroticism with *r*_s_ = −0.21. Furthermore, we found a small association of NFC with the frequency of termination thoughts (*r*_s_ = −0.18). Together, these results clearly support the assumption that higher NFC goes along with increasing satisfaction with and less doubts about one's studies and thereby extend previous findings on associations between NFC and affective adjustment.

Similarly, we found negative associations between NFC and three reasons for termination thoughts: first, with the feeling not to belong. This might be either a side effect of enhanced satisfaction by enabling more integration with other people or refer to independent social-emotional effects of NFC (Bertrams and Dickhäuser, 2012). Alternatively, individuals may not tend to experience enhanced feelings of belonging with increasing NFC but may not consider them relevant to think about quitting their studies. The second negatively associated reason was a lack of motivation: As stable motivational tendency to invest cognitive effort, NFC is likely to enhance a person's motivation to engage in the cognitive challenges involved in higher education. Third, NFC negatively correlated with a subjectively experienced shortage of relations between theory and practice. As higher NFC promotes an elaborated processing of information, one could imagine that higher NFC will promote the tendency to build ideas of such relations autonomously if lectures fail to present them explicitly. Alternatively, individuals with higher NFC may appreciate theoretical inputs more and NFC may influence how people evaluate the relation between theory and practice. In sum, the associations of NFC with termination thoughts and satisfaction equally point to its relevance for the way in which students experience their studies and at its meaning to outcomes beyond performance measures like grades. Taking into account the low frequency of statements for some categories, our results for reasons to think about quitting/changing one's studies should be taken as first exploratory results and need to be replicated.

Summarizing, the current results highlight the importance of NFC for affective processes and outcomes. They underscore the necessity to increase research activities on affective implications of NFC, and to examine the underlying processes or variables of these associations.

### How NFC predicts success in university

We originally assumed that NFC and broader personality traits would positively impact on both school and university GPA as well as study satisfaction, with the latter also being influenced by university GPA. Furthermore, we expected termination thoughts to arise from both lower university GPA and lower study satisfaction. Interestingly, despite bivariate relationships between NFC and Conscientiousness with university GPA as well as between NFC and school GPA, these relationships did not or not fully emerge in the comprehensive path model. Instead, the most prominent way in which NFC had an impact was a direct path to study satisfaction and via this variable on termination thoughts. Thus, the present results suggest a role of NFC on academic success primarily by way of its role in modulating study satisfaction that in turn reduces the likelihood of termination thoughts.

This finding is somewhat contradictory to the body of evidence that suggests a direct influence of NFC on academic success as indicated by school or university grades. However, these previous studies mainly only investigated bivariate relationships. Considering further modulating or, more precisely in the present context, mediating factors such as study satisfaction may aid in gathering a more precise insight in the complex role of NFC in academic contexts. Specifically, the present results highlight an often underestimated aspect of NFC, which is not only defined by the intrinsic motivation to but also by the *enjoyment* of effortful cognitive endeavors—with university studies certainly being one exemplar of the latter. Quite strikingly, university GPA had merely no effect on termination thoughts, while study satisfaction did and did so partly based on the individual level of NFC. Thus, one conclusion that can be drawn from the present findings is that in cognitively challenging situations, NFC has an impact on positive outcomes not or not only because of a higher motivation to master these challenges, but also because of the positive appraisal of (dealing with) these situations.

Our findings are in line with previous research that reported links of NFC to life satisfaction (Coutinho and Woolery, 2004) and to job satisfaction (Park et al., 2008) and suggest that NFC has implications for study satisfaction as well. With regard to the few findings on NFC and aspects of emotional adaptation (e.g., Bertrams and Dickhäuser, 2012), the current results underscore the notion that the relationship of NFC with affective variables is not limited to basic resources and general emotional states, but also translates into important life outcomes such as satisfaction in academic contexts. This is all the more relevant given the aforementioned evidence that satisfied students also tend to be the ones who perform better (Judge et al., 2001) and that dissatisfaction can—regardless of academic performance—limit the likelihood to graduate and to subsequently work in the respective field of expertise (e.g., Hellman, 1997). Summarizing, the present results highlight the role of NFC as an important resource in academic contexts by way of its affective implications.

### Limitations and future research

Our study provides a differentiated view on how NFC predicts success in university and highlights its importance in modulating study satisfaction. Future studies should follow up on these findings by including others' perspectives, objective performance assessments, and longitudinal designs in order to avoid reliance on retrospective measures or subjective estimations only. Furthermore, our measure of satisfaction with one's studies that resulted from an integration of existing scales should be further validated against other approaches to assess study satisfaction. Also, it may be worthwhile to distinguish between the role of NFC in study satisfaction in different types of study programs, i.e., programs that are more oriented toward basic science in comparison to programs that are more focused on applied sciences. Similarly, future studies could systematically examine differences in major subjects and intended degrees (bachelor vs. master). We encourage prospective research to extend our research by following its comprehensive perspective and by deepening the understanding of our results.

## Author contributions

JG and AnS conceived and designed the study, organized and supervised data collection, and pre-processing. JG and AlS analyzed the data, all three authors wrote parts of the manuscript and gave final approval of the manuscript to be published.

### Conflict of interest statement

The authors declare that the research was conducted in the absence of any commercial or financial relationships that could be construed as a potential conflict of interest.

## Footnotes

^1^These criteria include a sample of healthy adults, voluntary attendance, noninvasive measures, no deception, appropriate physical, and mental demands on subjects.

^2^The finally reported analyses do not include the three best grades as measure of success because of a large overlap with university GPA (*r*_s_ = 0.73).

^3^In the first version of this manuscript, three items aiming at individual confidence referring to study-specific demands were also included in the instrument. As a result of the review process, they are no more part of the study satisfaction scale in order to get a less heterogeneous measure. The main results as well as the internal consistency of the 12- and 15-item version were largely comparable.
