# Corrigendum: Cognitive Investments in Academic Success: The Role of Need for Cognition at University

**DOI:** 10.3389/fpsyg.2017.01272

**Published:** 2017-07-21

**Authors:** Julia Grass, Alexander Strobel, Anja Strobel

**Affiliations:** ^1^Personality Psychology and Assessment, Department of Psychology, Chemnitz University of Technology Chemnitz, Germany; ^2^Differential and Personality Psychology, Department of Psychology, Technische Universität Dresden Dresden, Germany

**Keywords:** need for cognition, academic success, satisfaction with one's studies, investment traits, academic performance

In the original article, we neglected to include the funder German Research Foundation/DFG and the Chemnitz University of Technology, who accepted to fund our publication. Therefore, the following statement should be added to our article:

“The publication costs of this article were funded by the German Research Foundation/DFG and the Chemnitz University of Technology in the funding program Open Access Publishing.”

The authors apologize for this error and state that this does not change the scientific conclusions of the article in any way.

## Conflict of interest statement

The authors declare that the research was conducted in the absence of any commercial or financial relationships that could be construed as a potential conflict of interest.

