# Supplementary material for: Cognitive Investments in Academic Success: The Role of Need for Cognition at University
Source: Front Psychol. 2017 May 16;8:790. doi: 10.3389/fpsyg.2017.00790 (PMC5432647; doi:10.3389/fpsyg.2017.00790)
Supplement: Supplementary file 1 [file Table1.docx]

**Appendix**

Table A1

*Items to Measure Satisfaction with One’s Studies*

| **German** | **English** |
| --- | --- |
| 1. Ich studiere gern. | 1. I like to study. |
| 1. Das Studium macht mir viel Spaß. | 1. I really enjoy to study. |
| 1. Insgesamt bin ich mit meiner jetzigen Studienwahl zufrieden. | 1. In general, I am satisfied with my current choice of study. |
| 1. Zu Studienbeginn freue ich mich wieder auf die Veranstaltungen. | 1. At the beginning of a semester, I look forward to joining the courses. |
| 1. Es ist für mich von großer persönlicher Bedeutung, gerade dieses Fach studieren zu können. | 1. Studying especially this subject has a great personal meaning to me. |
| 1. Im Vergleich zu anderen mir sehr wichtigen Dingen (Hobbies, soziale Beziehungen) messe ich meinem Studium eine große Bedeutung bei. | 1. Compared to other things that are important to me (leisure activities, social relations), my studies are of great importance for me. |
| 1. Ich wünschte mir, dass die Studienbedingungen an der Universität besser wären. | 1. I wished the study conditions at my university would be better. (recoded) |
| 1. Ich habe mein jetziges Studium vor allem wegen der interessanten Studieninhalte gewählt. | 1. I have chosen my current studies mainly because of its interesting contents. |
| 1. Die Beschäftigung mit den Inhalten meines Studienfachs hat für mich wenig mit Selbstverwirklichung zu tun. | 1. To me, dealing with contents of my study subject has little to do with self-fulfillment. (recoded) |
| 1. Ich beschäftige mich auch unabhängig von den Prüfungsanforderungen intensiv mit den Inhalten meines Studienfaches. | 1. I am dealing with contents of my current study independently on the requirements of examinations. |
| 1. Ich fühle mich durch das Studium oft müde und angespannt. | 1. I am often feeling tired and tensed by my studies. (recoded) |
| 1. Ich kann mein Studium nur schwer mit anderen Verpflichtungen in Einklang bringen. | 1. I have difficulties to allign my studies with other duties. (recoded) |

*Note*. Within this study, the German items were used. English translation only for the purposes of this article.
